# Self-Sacrifice or Cowardice

**Published:** 1908-01-04

**Authors:** 


### Nursing Administration.

### SELF-SACRIFICE OR COWARDICE.

### THE QUESTION OF OVERWORK.

It is sadly too common to meet with people who are aged before their time, and who stand confessed the victims of overwork. Very often these are people who have unfortunately been forced into positions beyond their powers. Compelled to cope with duties too complex and arduous for their grasp, they have struggled along hopelessly perhaps for years, until the slow strain of conscious inadequacy has resulted in serious illness. The world is full of persons who fill quite inadequately important posts, but this kind of strain seldom arises except when it is associated with a sensitive conscience, and is far commoner in women than in men. Many hospital matrons break down simply and solely because, although they have been excellent nurses, they are incompetent to govern.

But if the issue be narrowed to those who break down, not because of the strain entailed by some want in themselves, but because those duties are too numerous and arduous to be carried through by one woman, it becomes an interesting question whether such an one is justified in allowing her health to be wrecked, and her future embittered even in the cause of an institution she loves. For this is the inevitable result of a process which may be witnessed, alas, in far too many institutions throughout the country. In fact, it may be doubted if ever before anything like the same strain has been the rule. It is easy to understand how this has arisen. A general wave of enthusiasm has passed over the training schools for nurses, which has resulted in the provision of better quarters, easier hours, wholesome recreation, excellent food, and increased rates of pay for nurses and probationers in training. All these things have added largely to the labours of the matron. The Nurses' Home requires supervision, the housekeeping demands more attention, the teaching is more arduous, and a far higher level of efficiency in every department is expected. With all this the position of the matron has by no means altered for the better. It is rare to find that the matron's salary has been increased, although her duties may have been doubled. It is quite certain that her powers of work cannot be doubled in proportion to the additional responsibilities imposed, but often one pair of hands is expected to get through what would supply ample occupation for three. Vainly contending against nature, the matron in such cases frequently toils on with a brave spirit until some slight reverse of health doubles the fatigues of her office, when the inevitable breakdown can no longer be deferred. Are we to honour this spirit, and deem such wrecked careers a fitting sacrifice to a good cause? We believe that the time is approaching when we shall repudiate such mistaken, if heroic, efforts, and shall deem it to be the duty of those concerned with the care of the sick to observe the laws of health in their own persons as well as in those under their care. Regarded dispassionately overwork is a bad blunder. It is a symptom of defective organisation, and where bad organisation exists the quality of the work must inevitably suffer. It is often very difficult in hospitals to fix the blame for overwork on the right individual. Sometimes it is the result of no one in particular, but has come about in consequence of small changes, whose significance from the point of view of work no one has perceived. Sometimes it results from ill-judged parsimony on the part of committees. More often it is a condition of affairs which passes as a matter of course in the busy atmosphere of a busy institution. The line of separation is, indeed, not always easy to discover between work up to the top of the powers, and work which goes much beyond them. In general it may be said that a post which demands exceptional physical strength —the power of dispensing, for instance, with reasonable hours of sleep, and reasonable moments of leisure—is a post which exposes its occupants to danger. In our opinion, those who find themselves in such posts should not, even if able for a term of years to stand the strain by reason of exceptional powers of mind and body, be content to hand it on to successors without making a vigorous effort to change its conditions. A matron who knows that the work she is doing is beyond her strength, and yet continues in office is preparing disaster for those who come after her. It may be that her committee reply to her representations in favour of additional assistance with the usual plea of "Want of means." Nothing can excuse the governors of institutions founded to heal the sick for a reckless parsimony, which piles on the officers chosen to carry out the objects of the charity duties which must eventually reduce them to the level of those they have set out to relieve. Therefore when subscribers and governors are indifferent, or unbelieving, the matron must take the matter into her own hands. She ought to prepare a report stating in what particulars the work is beyond her strength, and must continue to be beyond the ordinary powers of any successor. She ought to be prepared with suggestions for making the post one which could be filled adequately without undue strain. And then if her remonstrance is disregarded, she ought to have the courage of her opinions and go. Such a course demands far more courage than desperate acquiescence in a continuous whirl of worry. The reason which prevents its being followed is often highly creditable. It is a deep sense of the value of the work, and a fear of its suffering if the office falls into other hands. We believe this reasoning to be false and the self-sacrifice which perpetuates the system to be bad. It is not for the ultimate good of the charity that those who rule shall be weighed down with the incessant endeavour to triumph over fatigue of mind and body. It is the plain duty of the matron working under impossible conditions to rescue herself, her successor, and the charity she serves from inevitable disaster by the only instrument within her reach—retirement from office.

